# Protective Effect of Panaxynol Isolated from *Panax vietnamensis* against Cisplatin-Induced Renal Damage: In Vitro and In Vivo Studies

**DOI:** 10.3390/biom9120890

**Published:** 2019-12-17

**Authors:** Dahae Lee, Jaemin Lee, Kim Long Vu-Huynh, Thi Hong Van Le, Thi Hong Tuoi Do, Gwi Seo Hwang, Jeong Hill Park, Ki Sung Kang, Minh Duc Nguyen, Noriko Yamabe

**Affiliations:** 1 College of Korean Medicine, Gachon University, Seongnam 13120, Korea; pjsldh@naver.com (D.L.); jaemin.lee426@gmail.com (J.L.); seoul@gachon.ac.kr (G.S.H.); kkang@gachon.ac.kr (K.S.K.); 2 Faculty of Pharmacy, Ton Duc Thang University, Ho Chi Minh City 70000, Vietnam; vuhuynhkimlong@tdtu.edu.vn; 3 Faculty of Pharmacy, University of Medicine and Pharmacy at Ho Chi Minh City, Ho Chi Minh City 70000, Vietnam; levan@ump.edu.vn (T.H.V.L.); hongtuoid99@gmail.com (T.H.T.D.); 4 College of Pharmacy, Seoul National University, Seoul 151-742, Korea; hillpark@snu.ac.kr

**Keywords:** *Panax vietnamensis*, panaxynol, cisplatin-induced renal damage, reno-protective activity, cytotoxicity, MAPKs, caspase-3

## Abstract

Polyacetylenic compounds isolated from *Panax* species are comprised of non-polar C17 compounds, exhibiting anti-inflammatory, antitumor, and antifungal activities. Panaxynol represents the major component of the essential oils of ginseng. We investigated whether panaxynol isolated from *Panax vietnamensis* (Vietnamese ginseng, VG) could prevent cisplatin-induced renal damage induced in vitro and in vivo. Cisplatin-induced apoptotic cell death was observed by staining with annexin V conjugated with Alexa Fluor 488, and western blotting evaluated the molecular mechanism. Panaxynol at concentrations above 0.25 μM prevented cisplatin-induced LLC-PK1 porcine renal proximal tubular cell death. LLC-PK1 cells treated with cisplatin demonstrated an increase in apoptotic cell death, whereas pretreatment with 2 and 4 μM panaxynol decreased this effect. Cisplatin demonstrated a marked increase in the phosphorylation of c-Jun N-terminal kinase (JNK), P38, and cleaved caspase-3. However, pretreatment with 2 and 4 μM panaxynol reversed the upregulated phosphorylation of JNK, P38, and the expression of cleaved caspase-3. We confirmed that the protective effect of panaxynol isolated from *P. vietnamensis* in LLC-PK1 cells was at least partially mediated by reducing the cisplatin-induced apoptotic damage. In the animal study, panaxynol treatment ameliorated body weight loss and blood renal function markers and downregulated the mRNA expression of inflammatory mediators.

## 1. Introduction

The kidney is the main organ responsible for maintaining homeostasis in the body by regulating the volume and chemical composition of body fluids [1]. Thus, the kidney accumulates more toxic chemicals than other organs [2]. Studies have reported that 60% of all clinical nephrotoxicity cases are primarily attributed to anticancer drugs, particularly cisplatin [3]. Therefore, the incidence of nephrotoxicity may increase with the incidence of cancer, a leading cause of death with 18.1 million new cases and 9.6 million cancer deaths in 2018 [4]. This necessitates the discovery of alternatives that are effective but less toxic in cancer patients [5].

Ginsenosides are the major active constituents in the *Panax* species. Several studies have reported the protective effects of ginsenoside in damaged proximal tubular cells, a major site for cisplatin effects, and in animal models of cisplatin-induced renal damage [6]. In human embryonic kidney epithelial cells (HEK293) and mice, ginsenoside Rb3 reduced renal damage via the regulation of autophagy and inhibition of proximal tubular apoptosis [7]. Reportedly, ginsenosides Rh2, Re, and Rg5 prevent oxidative stress, inflammation, and apoptosis in cisplatin-induced renal damage in mice [8,9,10]. Furthermore, treatment with ginsenosides Rk3, Rh4, and Rd reduced cytotoxicity in the porcine renal proximal tubular cell line LLC-PK1 and improved the renal histology in cisplatin-induced acute kidney injury in rats [11,12,13]. Ginsenosides Rh3 and Rg3 can reduce apoptotic cell death in LLC-PK1 cells [14,15]. To date, the active ingredient research of ginseng has mainly focused on ginsenosides. Recently, with the development of various analytical techniques, a growing number of studies have investigated components other than ginsenosides.

The C17-polyacetylenes, which include panaxynol and its related epoxide panaxydol, have attracted interest due to their biological activities [16,17]. Panaxynol and panaxydol represent the two major polyacetylenes and are the major essential oil components of ginseng. Panaxynol and related polyenes have mainly demonstrated cytotoxic activity against several human tumor cell lines in vitro and in vivo [5,18,19]. Furthermore, panaxynol has exhibited anti-inflammatory and antifungal activities [20]. The anti-inflammatory activity of panaxynol has been reported in lipopolysaccharide (LPS)-stimulated macrophages, inhibiting the expression of inflammatory cytokines [21]. Moreover, the anti-inflammatory activity suppressed cyclooxygenase-2 (COX-2) immunoreactivity in dextran sulfate sodium (DSS)-induced colitis in mice and inhibited the expression of inducible nitric oxide synthase (iNOS) in interferon-γ (IFNγ)-stimulated macrophages [22]. Additionally, panaxynol has also demonstrated antioxidant activity. Panaxynol pretreatment reduced the oxidative stress induced by amyloid β-protein fragment 25–35 (Aβ25–35) in primary cultured rat cortical neurons [23]. In 3T3-L1 adipocytes, panaxynol reportedly inhibits the increased levels of reactive oxygen species (ROS) due to palmitic acid exposure [24].

However, the actions of panaxynol in cisplatin-induced renal damage are still unknown. Cisplatin results in nephrotoxicity by stimulating oxidative stress and inflammation, important determinants of this side effect [25]. Cisplatin-induced mitochondrial dysfunction enhances the generation of ROS due to the reaction of cisplatin with endogenous glutathione. In addition, the inflammatory response is linked to the cisplatin-induced renal tissue damage through the secretion of inflammatory cytokines such as tumor necrosis factor-α (TNF-α), interleukin 1 (IL-1), and IL-6 [26]. Natural products possessing potent antioxidant and anti-inflammatory properties are being evaluated against cisplatin-induced nephrotoxicity [27,28,29].

Although differing in cell types and concentrations, considering the antioxidant and anti-inflammatory properties, panaxynol may possess a renoprotective effect. Therefore, we explored the mechanisms involved in the protective effect of panaxynol against cisplatin-induced renal damage in vitro and in vivo.

## 2. Materials and Methods

### 2.1. Plant Material

Vietnamese ginseng (VG) was collected at Tra Linh farm, Quang Nam province in 2016. A voucher specimen was deposited in the herbarium of the College of Pharmacy, Seoul National University, Seoul, Korea (SNUP-2016-A-01). The VG roots were dried at 40–60 °C, and subsequently ground and sieved to obtain a powder.

### 2.2. HPLC Analysis of Panaxynol

Panaxynol was prepared at the concentration of 100 ppm in methanol (MeOH). A total of 150 mg of VG powder was extracted by sonication with 10 mL MeOH for 30 min at 40 °C. The solution was then filtered via 0.22 µm membrane filter prior to ultra performance liquid chromatography (UPLC) analysis. UPLC was performed on an ACQUITY UPLC H-Class system (Waters, Milford, MA, USA) equipped with photodiode array detector (PDA) detector (203 nm) and Phenomenex Gemini C18 (150 × 4.6 mm. i.d., 3 μm) (Phenomenex, Torrance, CA, USA) connected to Empower software. The separation was achieved with mobile phase of acetonitrile (A) and water (B) as follows: 0–8 min: 22–55% A; 8–20 min: 55–90% A; 22–27 min: 90–95% A; 27–30 min: 22% A; 30–35 min: 22% A. The column temperature was set at 30 °C. The HPLC flow rate was 0.65 mL/min. A total of 5 μL of sample solution was injected into the UPLC system.

### 2.3. Extraction and Isolation

VG dried powder (300 g) was extracted with 1000 mL of MeOH by sonication six times. The filtered MeOH extract was evaporated in vacuo to yield the MeOH total extract (120 g), which was suspended with water and partitioned between diethyl ether (Et_2_O) and water. The Et_2_O fraction was then evaporated in vacuo to yield the Et_2_O extract (4.2 g). The Et_2_O fraction was chromatographed on a silica gel column using a stepwise gradient elution with a mixture of n-hexane and ethyl acetate (30:1→15:1→8:1→4:1→1:1) to yield five sub-fractions (Et1–Et5). Sub-fraction Et3 (1.2 g) was further purified using a silica gel column and elution with n-hexane and ethyl acetate (8:1) to yield panaxynol (600 mg) as a yellow liquid. The structure of panaxynol was confirmed through comparison with published data of ^1^H and ^13^C NMR [30].

### 2.4. Cell Culture

The porcine renal proximal tubular cell line LLC-PK1 was purchased from the American Type Culture Collection (Rockville, MD, USA). Cells were cultured in Dulbecco′s modified Eagle medium (DMEM) (Cellgro, Manassas, VA, USA) containing 10% FBS, 1% penicillin/streptomycin (Invitrogen Co., Grand Island, NY, USA), and 4 mM l-glutamine. To maintain cell growth, cells were maintained in 5% CO_2_ in humidified air and at 37 °C.

### 2.5. Cell Viability Assay

To detect the cytotoxicity of cisplatin (Sigma, St. Louis, MO, USA) and evaluate the protective effect of panaxynol in cisplatin-induced cytotoxicity in LLC-PK1 cells, the cells were seeded in 96-well culture plates (10,000 cells per well) and incubated for 24 h. The cells were pretreated with panaxynol at a wide range of concentrations (0.0625, 0.125, 0.25, 0.5, 1, 2, 4 μM) for 2 h. Next, 25 μM cisplatin was added to each well, except for the control (0.5% DMSO) wells. After incubation for 24 h, cell viability was determined using the Ez-Cytox (Daeil Lab Services; Seoul, Korea) assay in accordance with the manufacturer’s instructions. The mean optical density (OD, absorbance) of each well at 450 nm was measured using a microplate reader (PowerWave XS; Bio-Tek Instruments, Winooski, VT, USA) and used to calculate the percentage of cell viability (percentage of cell viability = (A_treatment_ − A_blank_)/(A_control_ − A_blank_) × 100% (where, A = absorbance)).

### 2.6. Quantification of Apoptosis with Image-Based Cytometric Assay

An image-based cytometric assay was performed to observe the effect of panaxynol on cisplatin-induced apoptosis in LLC-PK1 cells. The cells were seeded in 6-well culture plates (200,000 cells per well) and incubated for 24 h. The cells were pretreated with panaxynol at 2 and 4 μM for 2 h, and then 25 μM cisplatin was added to each well, except for the control (0.5% DMSO) wells. After 24 h, harvested cells were suspended at 500,000 cells/100 μL in binding buffer. The cell suspension in 100 μL aliquot was incubated with 5 μL of annexin V Alexa Fluor 488 for 30 min in the dark at room temperature. Annexin V-positive-stained cells were measured using a Tali image-based cytometer (Invitrogen, Temecula, CA, USA).

### 2.7. Western Blot Analysis

The expression levels of c-Jun N-terminal kinase (JNK), P38, and cleaved caspase-3 were detected using the western blotting method. Cells were treated as described in the above image-based cytometric assay and harvested on ice. According to the manufacturer’s instructions, cells were lysed using the radioimmunoprecipitation assay buffer (RIPA buffer) (Cell Signaling Technology Inc., Beverly, MA, USA) supplemented with 1 mM phenylmethylsulfonyl fluoride (PMSF). The protein concentrations were quantified using the Pierce BCA protein assay kit. An equal amount of protein (20 μg) per lane was separated by electrophoresis in a 10% sodium dodecyl sulfate-polyacrylamide gel and transferred onto PVDF transfer membranes. Specific proteins on the membrane were detected by epitope-specific primary antibodies to JNK, P-JNK, P38, P-P38, cleaved caspase-3, GAPDH, and horseradish peroxidase (HRP)-conjugated anti-rabbit antibodies, followed by enhanced chemiluminescence (ECL) advance western blotting detection reagents and visualized using a FUSION Solo chemiluminescence system (PEQLAB Biotechnologie GmbH, Erlangen, Germany).

### 2.8. Cisplatin-Induced Nephrotoxicity in Mice

The animal experiment was approved by the Animal Care and Use Committee of Gachon University (GIACUC-R2019026). Seven-week-old male C57BL/6 mice were used to evaluate cisplatin-induced nephrotoxicity. The animals were housed at 23 ± 2 °C, 55 ± 5% humidity, with a 12 h light/dark cycle, and free access to water and food. Acute kidney injury was induced by a single intraperitoneal injection of cisplatin at a dose of 16 mg/kg in saline (day 0). *N*-acetyl-l-cysteine (NAC, 1000 mg/kg) and panaxynol (10 and 50 mg/kg) were orally administered once daily for 5 days. NAC (Sigma, St. Louis, MO, USA), a well known antioxidant, was used as a reference drug because there are many comparative experimental data using the NAC against cisplatin-induced nephrotoxicity using both in vitro and in vivo experimental models [31,32,33]. Five mice were allotted to the normal group and eight mice were allotted to each of the cisplatin-injected groups (cisplatin, NAC, panaxynol). Panaxynol and NAC were dissolved in 0.5% methylcellulose solution, and oral administration began 1 day before the cisplatin injection. The body weight was measured daily for 4 days (day 0–3). Three days after the cisplatin injection (day 3), blood samples were collected by performing a cardiac puncture under light anesthesia using diethyl ether, followed by the dissection and isolation of kidneys.

### 2.9. Determination of Serum Creatinine and Blood Urea Nitrogen

The serum was obtained from whole blood by centrifugation at 1200× *g* for 20 min. Serum level of creatinine and blood urea nitrogen (BUN) were measured using a commercial kit (YD diagnostics, Yongin, Korea) according to the manufacturer’s instructions.

### 2.10. Real-Time PCR for Cyclooxygenase-2, Monocyte Chemoattractant Protein-1, and Hypoxanthine Phosphoribosyltransferase-1

Total RNA was extracted from the kidney using the NuActor kit (Ugenecell, Chuncheon, Korea) according to the manufacturer’s instructions. Reverse transcription was performed using the RevertAid First Strand cDNA Synthesis kit (Fermentas, Waltham, MA, USA) and random primers, followed by real-time PCR. Real-time PCR amplification of cyclooxygenase-2 (COX-2), monocyte chemoattractant protein-1 (MCP-1), and hypoxanthine phosphoribosyltransferase-1 (HPRT1) was performed using the Power SYBR Green PCR Master Mix (Applied Biosystems, Foster City, CA, USA). The following primer pairs were used: COX-2, forward 5′-CTGGAACATGGACTCACTCAGTTTG-3′ and reverse 5′-AGGCCTTTGCCACTGCTTGTA-3′; MCP-1, forward 5′-GTCCCTGTCATGCTTCT GG-3′ and reverse 5′-GCGTTAACTGCATCTGGCT-3′; HPRT1, forward 5′-GATTAGCGATGAT GAACCAGGTT-3′ and reverse 5′-CCTCCCATCTCCTTCATGACA-3′. The PCR conditions consisted of 10 s at 95 °C, followed by 40 cycles of 5 s at 95 °C, and 20 s at 60 °C using QuantStudio 3 (Thermo Fischer Scientific, Waltham, MA, United States). Target mRNA levels were normalized to those of HPRT1 as an internal control in each sample. The results are expressed as the ratio relative to the normal group average.

### 2.11. Statistical Analysis

Statistical significance was determined using one-way analysis of variance (ANOVA), and multiple comparisons were performed using a Bonferroni correction. *P*-values of less than 0.05 indicated statistical significance. All analyses were performed using SPSS Statistics ver. 19.0 (SPSS Inc., Chicago, IL, USA).

## 3. Results

### 3.1. Protective Effect of Panaxynol Isolated from Panax vietnamensis on Cisplatin-Induced LLC-PK1 Cell Death

**Figure 1:**
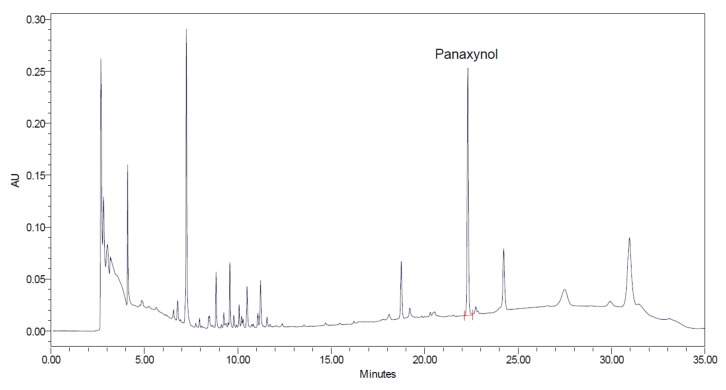
HPLC chromatogram of Vietnamese ginseng (VG) methanol (MeOH) extract. AU: absorbance unit.

**Figure 2:**
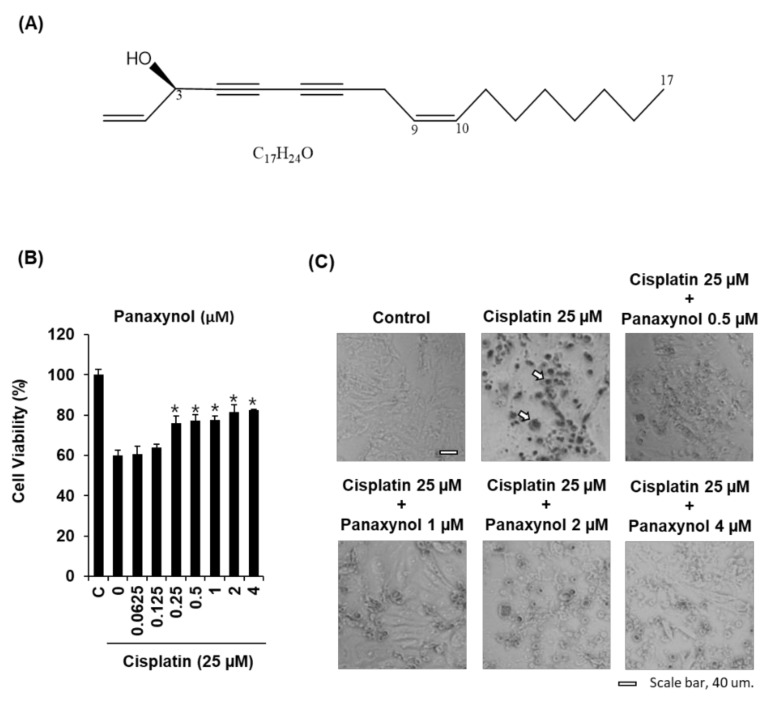
Protective effect of panaxynol isolated from *Panax vietnamensis* on the cisplatin-induced cytotoxicity in LLC-PK1 cells. (**A**) Chemical structure of panaxynol. (**B**) Cell viability in cisplatin-treated cells in the presence or absence of panaxynol (mean ± SD, * *p* < 0.05 as compared to the cisplatin-treated group). (**C**) Morphological changes confirmed under phase-contrast microscopy. C: control group treated with medium containing 0.5% DMSO. White scale bar, 40 um.

In the present study we isolated panaynol from VG, and it was analyzed with retention times of 22.3 min (Figure 1). To determine the non-toxic dose ranges of panaxynol, we assessed the cytotoxic effect of various concentrations of panaxynol on LLC-PK1 cells. As shown in Figure S1, panaxynol at 1, 2 and 4 μM show no toxic effects. To evaluate the protective effect of panaxynol (Figure 2A), LLC-PK1 cells were exposed to 25 μM cisplatin in the presence or absence of panaxynol for 24 h. As shown in Figure 2B, no protective effect on LLC-PK1 cells was observed at panaxynol concentrations below 0.125 μM. However, the cisplatin-induced decrease in cell viability to 59.94 ± 2.72% was increased to 75.95 ± 3.73%, 77.24 ± 2.93%, 77.47 ± 2.24%, 81.40 ± 3.60%, and 82.63 ± 0.35%, after pretreatment with 0.25, 0.5, 1, 2, and 4 μM of panaxynol, respectively. The cell morphology was confirmed using inverted phase-contrast microscopy under a 20× objective. Figure 2C revealed that the morphological shapes of the cisplatin-treated cells were detached from the surface and characterized by bubbling, cellular shrinkage, and deformation of cell bodies, indicating cell death. These changes were concentration-dependently suppressed by panaxynol. Thus, 2 and 4 µM of panaxynol were chosen as the highest optimal dose for further study.

### 3.2. Effect of Panaxynol Isolated from Panax vietnamensis on Cisplatin-Induced Apoptosis in LLC-PK1 Cells

**Figure 3:**
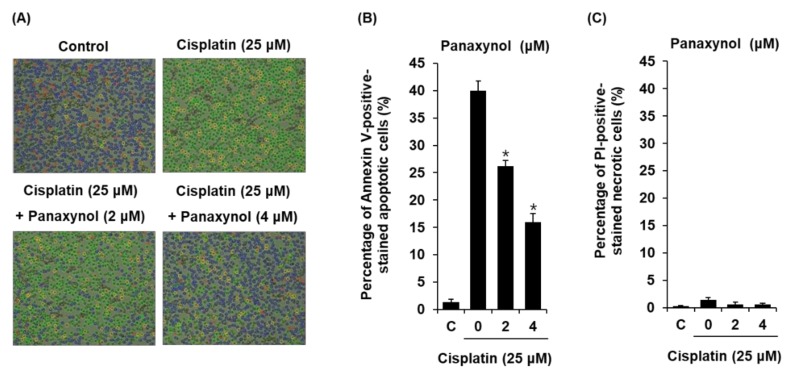
Effects of panaxynol isolated from *Panax vietnamensis* on apoptosis in LLC-PK1 cells exposed to cisplatin scored by an image-based cytometric assay. (**A**) Representative images for apoptosis detection and (**B**) percentage of annexin V-positive-stained apoptotic cells (40x magnification) (**C**) and PI-positive-stained necrotic cells (mean ± SD, * *p* < 0.05 as compared to the cisplatin-treated group) after treatment with cisplatin (25 μM) in the presence or absence of panaxynol. C: control group treated with medium containing DMSO (0.5% DMSO).

To explore whether panaxynol could decrease cisplatin-induced apoptosis, LLC-PK1 cells were exposed to 25 μM cisplatin in the presence or absence of 2 and 4 µM of panaxynol for 24 h and stained with annexin V conjugated with Alexa Fluor 488 to identify apoptotic cells and propidium iodide to identify necrotic cells. As shown in Figure 3A, cisplatin-treated cells displayed a highly increased green fluorescence indicating apoptotic cells stained with annexin V conjugated with Alexa Fluor 488 compared to control cells. However, pretreatment with 2 and 4 μM of panaxynol reduced the green fluorescence. As shown in Figure 3B, the percentage of apoptotic cells increased by 39.96 ± 1.76% after treatment with 25 μM cisplatin and decreased following treatment with 2 and 4 μM panaxynol to 26.23 ± 1.51% and 15.96 ± 1.53%, respectively. These results suggest that panaxynol prevented cisplatin-induced apoptotic cell death through its anti-apoptotic activity.

### 3.3. Effect of Panaxynol Isolated from Panax vietnamensis on Expression Levels of JNK, P38, and Cleaved Caspase-3 in Cisplatin-Treated LLC-PK1 Cells

**Figure 4:**
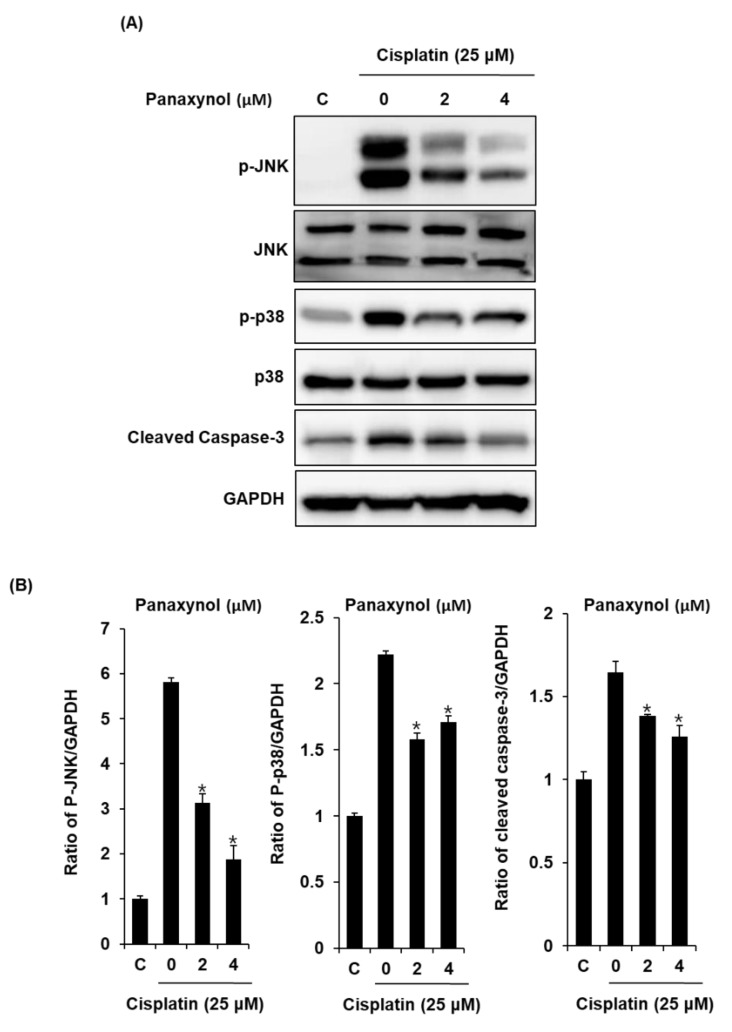
Effect of panaxynol isolated from *Panax vietnamensis* on expression levels of c-Jun N-terminal kinase (JNK), P38, and cleaved caspase-3 in LLC-PK1 cells exposed to cisplatin by western blotting. (**A**) Expressions of JNK, P38, and cleaved caspase-3 and (**B**) densitometric quantifications (mean ± SD, * *p* < 0.05 as compared to the cisplatin-treated group) after treatment with cisplatin (25 μM) in the presence or absence of panaxynol. C: control group treated with medium containing 0.5% DMSO.

Western blot analysis was performed to evaluate the molecular mechanism, focusing on JNK, P38, and cleaved caspase-3. As shown in Figure 3, treatment with 25 μM cisplatin increased the phosphorylation of JNK, P38, and the expression level of cleaved caspase-3. However, pretreatment with 2 and 4 μM panaxynol reversed the upregulated phosphorylation of JNK, P38, and expression of cleaved caspase-3 (Figure 4).

### 3.4. Effects of Panaxynol Isolated from Panax vietnamensis on Cisplatin-Induced Nephrotoxicity in Mice

**Figure 5:**
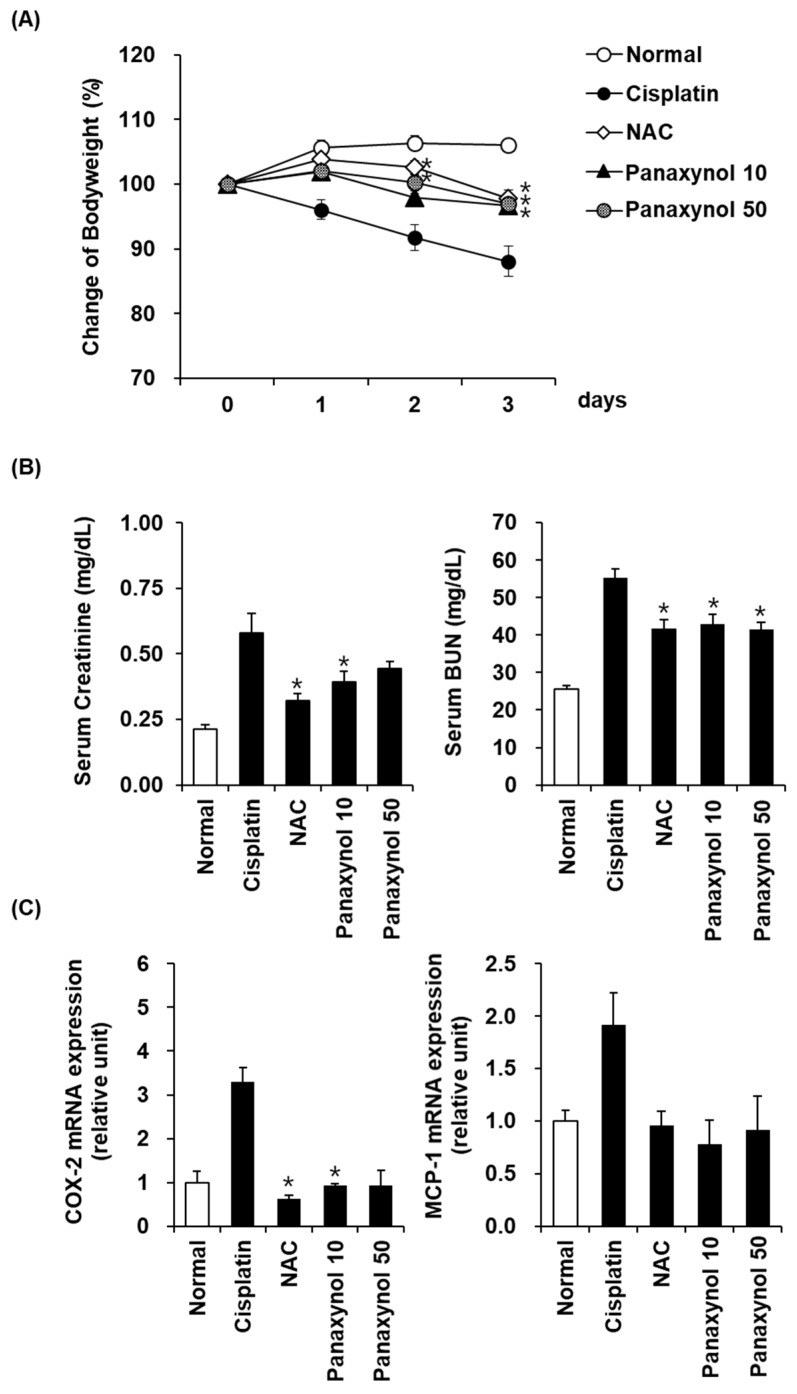
Effects of panaxynol isolated from *Panax vietnamensis* on cisplatin-induced nephrotoxicity in mice. (**A**) Bodyweight loss in cisplatin-induced nephrotoxicity in mice. (**B**) Serum creatinine and blood urea nitrogen (BUN) levels in cisplatin-induced nephrotoxicity in mice. (**C**) Cyclooxygenase-2 (COX-2) and monocyte chemoattractant protein-1 (MCP-1) mRNA expression in the kidney. Data are presented as the mean ± SEM. * *p* < 0.05 as compared to the cisplatin group. NAC: *N*-acetyl-l-cysteine; panaxynol 10 and 50: panaxynol 10 mg/kg and 50 mg/kg.

To investigate the effect of panaxynol on cisplatin-induced nephrotoxicity, alterations in body weight, renal function markers (creatinine and BUN), and inflammatory mediators (COX-2 and MCP-1) were evaluated in the mouse disease model. The cisplatin injection resulted in severe loss of bodyweight in mice. However, panaxynol (10 and 50 mg/kg) treatment significantly attenuated the loss of bodyweight 3 days after the cisplatin injection (Figure 5A). Moreover, the elevated serum creatinine (normal: 0.21 ± 0.02 mg/dL, cisplatin: 0.58 ± 0.08 mg/dL, NAC: 0.32 ± 0.03 mg/dL, panaxynol 10: 0.39 ± 0.04 mg/dL, panaxynol 50: 0.43 ± 0.03 mg/dL) and BUN (normal: 25.55 ± 0.88 mg/dL, cisplatin: 55.38 ± 2.38 mg/dL, NAC: 41.64 ± 2.40 mg/dL, panaxynol 10: 42.90 ± 2.63, panaxynol 50: 41.60 ± 1.77 mg/dL) levels were significantly decreased following panaxynol treatment (Figure 5B). It is well known that cisplatin-induced nephrotoxicity is associated with inflammation [33]. Therefore, we assessed the expression of COX-2 and MCP-1 mRNA as inflammatory markers in the kidney. Consistent with the improved serum creatinine and BUN, upregulated COX-2 mRNA expression was significantly downregulated following treatment with panaxynol (Figure 5C; normal: 1.00 ± 0.26, cisplatin: 3.29 ± 0.34, NAC: 0.63 ± 0.08, panaxynol 10: 0.93 ± 0.05, panaxynol 50: 94 ± 0.33). Although without statistical significance, MCP-1 mRNA expression demonstrated a tendency toward downregulation in the panaxynol treated group (Figure 5C; normal: 1.00 ± 0.10, cisplatin: 1.91 ± 0.31, NAC: 0.96 ± 0.14, panaxynol 10: 0.78 ± 0.23, panaxynol 50: 0.91 ± 0.32).

## 4. Discussion

Although many studies have reported the protective effects of ginsenosides in cisplatin-induced renal damage, the protective effect of panaxynol remains unknown. Previous studies have suggested that cisplatin damages the proximal tubular cells using molecular mechanisms related to the MAPK signaling and apoptotic pathways. In the MAPK signaling pathway, JNK and P38 are the main mediators of apoptotic cell death in the proximal tubular cells in response to cisplatin [34]. Cleaved caspase-3 with JNK and P38 are regarded as important therapeutic targets against cisplatin-induced damage in the proximal tubular cells [35]. Several studies have demonstrated that ginsenosides can attenuate the cisplatin-induced upregulation of phosphorylation of JNK, P38, and the expression levels of cleaved caspase-3 in proximal tubule cells. Ginsenosides Rb1 [7], Rh2 [8], Rh3 [14], and Rg3 [15,36] provide protection in cisplatin-induced tubular cell damage by suppressing the activation of caspase-3 that activates the extrinsic and intrinsic apoptotic pathways. In addition, ginsenosides Rh3 [14] and Rg3 [15] attenuate cisplatin-induced tubular cell damage by inhibiting phosphorylation of JNK and P38. In this study, we investigated the effects of panaxynol isolated from *P. vietnamensis* on cisplatin-induced cytotoxicity in LLC-PK1 cells. In the present study, cisplatin-induced LLC-PK1 cell death was suppressed by panaxynol. To further investigate the detailed mechanism, staining with annexin V conjugated with V Alexa Fluor 488 was performed. Cisplatin exposure increased the apoptotic cell death in LLC-PK1 cells. In contrast, panaxynol protected LLC-PK 1 cells from the cisplatin-induced apoptosis. Although various proteins were involved in cisplatin-induced apoptosis, we confirmed the protective effect of panaxynol in apoptotic cell death in LLC-PK1 cells using JNK, P38, and cleaved caspase-3. Cisplatin increased the phosphorylation of JNK, P38, and the expression level of cleaved caspase-3, and pretreatment with panaxynol effectively suppressed these observed upregulations. Hence, panaxynol inhibited apoptosis by downregulating the phosphorylation of JNK, P38, and the expression levels of cleaved caspase-3 in cisplatin-treated cells, contributing to the protective effect of panaxynol in cisplatin-induced LLC-PK1 cell death.

In the in vivo study, the oral administration of panaxynol ameliorated the cisplatin-induced kidney injury. Serum creatinine and BUN are the most commonly used biomarkers of renal dysfunction. Cisplatin is known to demonstrate toxic effects on the renal tubule, vasculature, and glomeruli. It results in structural damage to the kidney and consequently increases creatinine and urea in blood [37,38,39]. Panaxynol treatment reduced the serum creatinine and BUN in mice with cisplatin-induced kidney injury. Several reports have indicated that various inflammatory factors such as TNF-α, IL-6, MCP-1, and COX-2 are upregulated in cisplatin-induced nephrotoxicity in both humans and rodents [40,41]. The panaxynol-treated group exhibited significantly decreased COX-2 mRNA expression and slightly decreased MCP-1 mRNA expression in the kidneys compared to the cisplatin alone group. Collectively, these results indicate that panaxynol possesses a protective role in cisplatin-induced renal toxicity in mice and LLC-PK1 cells.

Panaxynol is a polyacetylene compound, which demonstrated renoprotective effects at low doses. The triple bonds in the structure could explain the ROS scavenging effects of panaxynol. Therefore, the kidney cells treated with this polyacetylene could possibly alleviate the oxidative stress induced by cisplatin. Additionally, panaxynol previously reported a strong antiproliferative effect [19,42]. The synergetic effect of the nephroprotective and antiproliferative effect of panaxynol could be an exciting combination in cancer patients, reducing the tumor as well as protecting the kidneys from cisplatin-induced injury. However, the potential clinical value of a panaxynol to protect against the cisplatin-induced nephrotoxicity will depend on its drug solubility, oral absorption, intestinal membrane permeability, and metabolism. Thus, further in vivo bioavailability studies will be needed.

## 5. Conclusions

This study, for the first time, confirmed the effect of panaxynol isolated from *P. vietnamensis* in cisplatin-induced renal damage in vitro and in vivo. Panaxynol reported a protective effect against cisplatin-induced LLC-PK1 cell death. We elucidated that this action was due to the antiapoptotic effects mediated through the downregulation of JNK, P38, and cleaved caspase-3, providing a better understanding of the pharmacological mechanisms of panaxynol in the treatment of cisplatin-induced nephrotoxicity.

## Supplementary Materials

The following are available online at https://www.mdpi.com/2218-273X/9/12/890/s1 Figure S1. Effect of panaxynol isolated from *Panax vietnamensis* in LLC-PK1 cells. C: control group treated with 0.5% DMSO.
