# Supplementary material for: Protective Effect of Panaxynol Isolated from Panax vietnamensis against Cisplatin-Induced Renal Damage: In Vitro and In Vivo Studies
Source: Biomolecules. 2019 Dec 17;9(12):890. doi: 10.3390/biom9120890 (PMC6995609; doi:10.3390/biom9120890)
Supplement: Supplementary file 1 [file biomolecules-09-00890-s001.pdf]

Supplementary Materials

Protective effect of panaxynol isolated from *Panax vietnamensis* against cisplatin-induced renal damage: *in vitro* and *in vivo* studies

Dahae Lee[1,#], Jaemin Lee[1,#], Kim Long Vu-Huynh[2], Thi Hong Van Le[3], Thi Hong Tuoi Do[3], Gwi Seo Hwang[1], Jeong Hill Park[4], Ki Sung Kang[1], Minh Duc Nguyen[2,*], Noriko Yamabe[1,*]

[1] College of Korean Medicine, Gachon University, Seongnam 13120, Korea; ; pjsldh@naver.com (D.L.); jaemin.lee426@gmail.com (J.L.); seoul@gachon.ac.kr (G.S.H.); kkang@gachon.ac.kr (K.S.K.)

[2] Faculty of Pharmacy, Ton Duc Thang University, Ho Chi Minh City 70000, Vietnam; vuhuynhkimlong@tdtu.edu.vn (K.L-V.H); nguyenminhduc@tdtu.edu.vn (M.D-N)

[3] Faculty of Pharmacy, University of Medicine and Pharmacy at Ho Chi Minh City, Ho Chi Minh City 70000, Vietnam; levan@ump.edu.vn (T.H.V.L.); hongtuoid99@gmail.com (T.H.T.D.)

[4] College of Pharmacy, Seoul National University, Seoul 151-742, Korea; hillpark@snu.ac.kr (JH-P)

[#] These authors contributed equally to the work described in this study.

* Correspondence: norikoy@gachon.ac.kr; Tel.: +82-31-750-5402 (N. Yamabe); nguyenminhduc@tdtu.edu.vn, Tel +84-90 898 8820 (M.D. Nguyen)

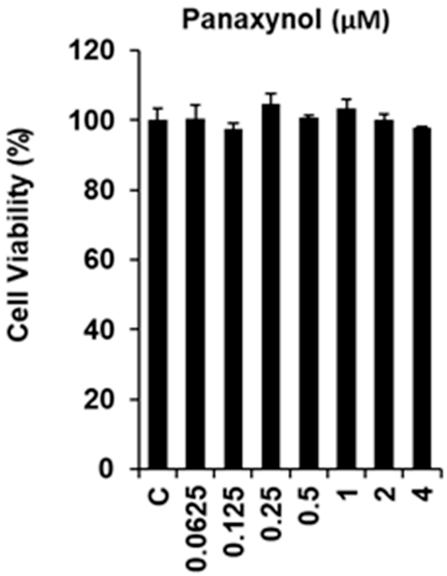

Figure S1. Effect of panaxynol isolated from *Panax vietnamensis* in LLC-PK1 cells. C: control group treated with 0.5% DMSO.
